# Bis[μ-1,1′-methyl­enebis(1*H*-imidazole)-κ^2^ *N* ^3^:*N* ^3′^]bis­[dichloridocobalt(II)]

**DOI:** 10.1107/S1600536811010610

**Published:** 2011-03-26

**Authors:** Miao Feng, Huai-Feng Mi, Tong-Liang Hu

**Affiliations:** a Biochemical Section of the Key Laboratory of Functional Polymer Materials, The Ministry of Education of China, Chemical School of Nankai University, 300071 Tianjin, People’s Republic of China; b Department of Chemistry, Nankai University, Tianjin 300071, People’s Republic of China

## Abstract

The title compound, [Co_2_Cl_4_(C_7_H_8_N_4_)_2_], contains a dinuclear complex molecule in which each Co^II^ atom is tetra­hedrally coordinated by two N atoms and two chloride ions. The 1,1′-methyl­enebis(1*H*-imidazole) ligands adopt a bis-monodentate bridging mode linking two Co^II^ atoms.

## Related literature

For background to the design and synthesis of new organic–inorganic hybrid materials, see: Wang *et al.* (2007*a* ▶,*b* ▶). For a related structure, see: Wang *et al.* (2007*b* ▶). 
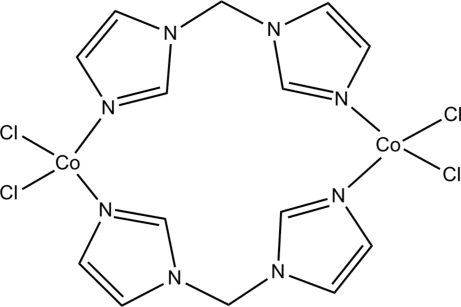


## Experimental

### 

#### Crystal data

[Co_2_Cl_4_(C_7_H_8_N_4_)_2_]
*M* *_r_* = 556.01
Monoclinic, 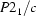

*a* = 8.7137 (17) Å
*b* = 8.7948 (18) Å
*c* = 14.560 (3) Å
β = 98.75 (3)°
*V* = 1102.8 (4) Å^3^
*Z* = 2
Mo *K*α radiation
μ = 2.01 mm^−1^
*T* = 293 K
0.3 × 0.3 × 0.3 mm

#### Data collection

Rigaku SCX-mini diffractometer
Absorption correction: multi-scan (*ABSCOR*; Higashi, 1995 ▶) *T*_min_ = 0.789, *T*_max_ = 1.0
11057 measured reflections
2501 independent reflections
2004 reflections with *I* > 2σ(*I*)
*R*_int_ = 0.040

#### Refinement

*R*[*F* ^2^ > 2σ(*F* ^2^)] = 0.040
*wR*(*F* ^2^) = 0.075
*S* = 1.13
2501 reflections
127 parameters
H-atom parameters constrained
Δρ_max_ = 0.30 e Å^−3^
Δρ_min_ = −0.33 e Å^−3^

### 

Data collection: *PROCESS-AUTO* (Rigaku, 1998 ▶); cell refinement: *PROCESS-AUTO*; data reduction: *CrystalStructure* (Rigaku/MSC, 2002 ▶); program(s) used to solve structure: *SHELXS97* (Sheldrick, 2008 ▶); program(s) used to refine structure: *SHELXL97* (Sheldrick, 2008 ▶); molecular graphics: *SHELXTL* (Sheldrick, 2008 ▶); software used to prepare material for publication: *publCIF* (Westrip, 2010 ▶).

## Supplementary Material

Crystal structure: contains datablocks I, global. DOI: 10.1107/S1600536811010610/jh2275sup1.cif

Structure factors: contains datablocks I. DOI: 10.1107/S1600536811010610/jh2275Isup2.hkl

Additional supplementary materials:  crystallographic information; 3D view; checkCIF report

## Figures and Tables

**Fig. 1.**
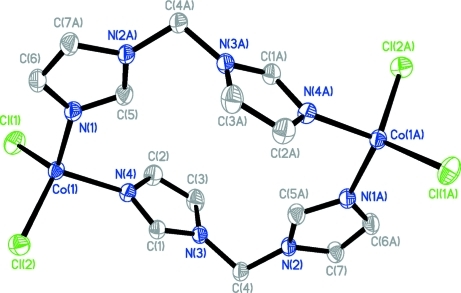
A view of the title compound.Ellipsoids are drawn at the 30% probability level.H atoms have been omitted for clarity. [Symmetry codes: (a) -x + 1, -y, -z + 1].

**Fig. 2.**
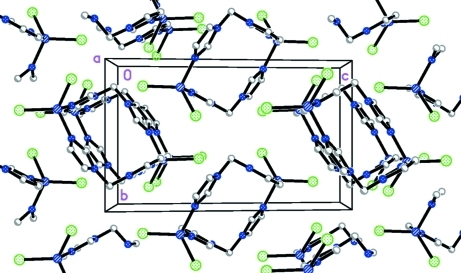
Packing diagram of the title compound.

## supplementary crystallographic information

### Comment

Currently, increasing attention has been attracted to the design and synthesis of new organic-inorganic hybrid materials (Wang *et al.*, 2007*a*). One of interesting strategies is using organic ligand to linking the metal salt. In our work the bridged ligand 1,1'-methylenedi-1*H*-imidazole (*L*) was selected to assemble novel organic-inorganic hybrid materials. Unexpectedly,the title compound, (I), was obtained with a dinuclear structure (Wang *et al.*, 2007*b*). As shown in Fig. 1, the crystal structure of (I) the two CoCl_2_ uints linked by two *L* ligands. In the complex the Co^II^ ion coordinated by two nitrogen atoms and and two Cl^-^ giving a tetrahedral geometry. The bond distances are normal range with of Co—N 2.020 (2) Å-2.029 (2)Å and Co—Cl 2.2478 (9) Å-2.2676 (9) Å. The *L* ligands all adopt a bis-monodentate bridging mode linking two Co^II^ atoms. atoms. The Cl^-^ anions coordinated to the Co^II^ atom in monodentate mode. By that way a diunclear complex was formed. The diunclear complex packing one by one in the soild state(Fig. 2).

### Experimental

In a typical synthesis, a mixture of Co(Cl)_2_.6H_2_O (0.05 mmol), 1,1'-methylenedi-1*H*-imidazole(0.05 mmol) and H_2_O (10 ml), was added to a 20 ml Teflon-lined reactor under autogenous pressure at 120 °C for 3 days. The resulting solution was slowly cooled to room temperature to yield single-crystal of the title compound.

### Refinement

All H atoms were positioned geometrically (C—H = 0.97Å and N—H = 0.90 Å) and allowed to ride on their parent atoms, with *U*_iso_(H) = 1.2*U*_eq_(parent atom).

### Crystal data

**Table:**

[Co_2_Cl_4_(C_7_H_8_N_4_)_2_]	*F*(000) = 556
*M**_r_* = 556.01	*D*_x_ = 1.674 Mg m^−^^3^
Monoclinic, *P*2_1_/*c*	Mo *K*α radiation, λ = 0.71073 Å
Hall symbol: -P 2ybc	Cell parameters from 9628 reflections
*a* = 8.7137 (17) Å	θ = 3.3–27.4°
*b* = 8.7948 (18) Å	µ = 2.01 mm^−^^1^
*c* = 14.560 (3) Å	*T* = 293 K
β = 98.75 (3)°	Block, red
*V* = 1102.8 (4) Å^3^	0.3 × 0.3 × 0.3 mm
*Z* = 2	

### Data collection

**Table:**

Rigaku SCX-mini diffractometer	2501 independent reflections
Radiation source: fine-focus sealed tube	2004 reflections with *I* > 2σ(*I*)
graphite	*R*_int_ = 0.040
ω scans	θ_max_ = 27.4°, θ_min_ = 3.3°
Absorption correction: multi-scan (*ABSCOR*; Higashi, 1995)	*h* = −11→11
*T*_min_ = 0.789, *T*_max_ = 1.0	*k* = −11→11
11057 measured reflections	*l* = −18→18

### Refinement

**Table:**

Refinement on *F*^2^	Primary atom site location: structure-invariant direct methods
Least-squares matrix: full	Secondary atom site location: difference Fourier map
*R*[*F*^2^ > 2σ(*F*^2^)] = 0.040	Hydrogen site location: inferred from neighbouring sites
*wR*(*F*^2^) = 0.075	H-atom parameters constrained
*S* = 1.13	*w* = 1/[σ^2^(*F*_o_^2^) + (0.024*P*)^2^ + 0.5905*P*] where *P* = (*F*_o_^2^ + 2*F*_c_^2^)/3
2501 reflections	(Δ/σ)_max_ = 0.001
127 parameters	Δρ_max_ = 0.30 e Å^−^^3^
0 restraints	Δρ_min_ = −0.33 e Å^−^^3^

### Special details

**Table:**

Geometry. All e.s.d.'s (except the e.s.d. in the dihedral angle between two l.s. planes) are estimated using the full covariance matrix. The cell e.s.d.'s are taken into account individually in the estimation of e.s.d.'s in distances, angles and torsion angles; correlations between e.s.d.'s in cell parameters are only used when they are defined by crystal symmetry. An approximate (isotropic) treatment of cell e.s.d.'s is used for estimating e.s.d.'s involving l.s. planes.
Refinement. Refinement of *F*^2^ against ALL reflections. The weighted *R*-factor *wR* and goodness of fit *S* are based on *F*^2^, conventional *R*-factors *R* are based on *F*, with *F* set to zero for negative *F*^2^. The threshold expression of *F*^2^ > σ(*F*^2^) is used only for calculating *R*-factors(gt) *etc*. and is not relevant to the choice of reflections for refinement. *R*-factors based on *F*^2^ are statistically about twice as large as those based on *F*, and *R*- factors based on ALL data will be even larger.

### Fractional atomic coordinates and isotropic or equivalent isotropic displacement parameters (Å^2^)

**Table:**

	*x*	*y*	*z*	*U*_iso_*/*U*_eq_	
Co1	0.71310 (4)	0.17519 (4)	0.29288 (2)	0.03334 (12)	
Cl1	0.67525 (10)	0.16673 (10)	0.13677 (5)	0.0560 (2)	
Cl2	0.86129 (8)	0.37006 (8)	0.35849 (5)	0.04539 (19)	
N1	0.7895 (3)	−0.0223 (3)	0.35434 (15)	0.0392 (5)	
N2	0.2068 (2)	0.2229 (2)	0.55419 (15)	0.0376 (5)	
N3	0.3271 (2)	0.2738 (2)	0.41927 (15)	0.0345 (5)	
N4	0.5028 (2)	0.2027 (2)	0.33439 (14)	0.0349 (5)	
C1	0.4789 (3)	0.2715 (3)	0.41130 (18)	0.0355 (6)	
H1	0.5570	0.3132	0.4546	0.043*	
C2	0.3577 (3)	0.1591 (4)	0.2905 (2)	0.0465 (7)	
H2	0.3378	0.1069	0.2344	0.056*	
C3	0.2496 (3)	0.2042 (4)	0.3417 (2)	0.0502 (8)	
H3	0.1429	0.1906	0.3272	0.060*	
C4	0.2601 (3)	0.3409 (3)	0.4956 (2)	0.0432 (7)	
H4A	0.1734	0.4054	0.4708	0.052*	
H4B	0.3373	0.4037	0.5328	0.052*	
C5	0.7094 (3)	−0.1052 (3)	0.4060 (2)	0.0441 (7)	
H5	0.6075	−0.0847	0.4139	0.053*	
C6	0.9327 (3)	−0.0932 (3)	0.3608 (2)	0.0459 (7)	
H6	1.0142	−0.0610	0.3312	0.055*	
C7	0.0639 (3)	0.2160 (3)	0.5834 (2)	0.0454 (7)	
H7	−0.0186	0.2829	0.5678	0.054*	

### Atomic displacement parameters (Å^2^)

**Table:**

	*U*^11^	*U*^22^	*U*^33^	*U*^12^	*U*^13^	*U*^23^
Co1	0.0356 (2)	0.0363 (2)	0.02950 (19)	0.00285 (16)	0.00930 (14)	0.00304 (16)
Cl1	0.0651 (5)	0.0732 (6)	0.0305 (4)	0.0026 (4)	0.0097 (3)	−0.0049 (4)
Cl2	0.0397 (4)	0.0467 (4)	0.0481 (4)	−0.0015 (3)	0.0016 (3)	−0.0035 (3)
N1	0.0421 (13)	0.0367 (13)	0.0423 (13)	0.0051 (11)	0.0175 (10)	0.0076 (11)
N2	0.0400 (13)	0.0338 (12)	0.0429 (14)	0.0061 (10)	0.0193 (11)	0.0092 (10)
N3	0.0344 (12)	0.0344 (12)	0.0364 (12)	0.0011 (10)	0.0114 (10)	0.0051 (10)
N4	0.0326 (12)	0.0410 (13)	0.0316 (12)	−0.0009 (10)	0.0065 (9)	−0.0017 (10)
C1	0.0325 (14)	0.0416 (15)	0.0328 (14)	−0.0034 (12)	0.0061 (11)	−0.0009 (12)
C2	0.0420 (16)	0.060 (2)	0.0367 (16)	−0.0092 (15)	0.0035 (13)	−0.0091 (14)
C3	0.0313 (15)	0.068 (2)	0.0510 (19)	−0.0085 (15)	0.0052 (14)	−0.0006 (16)
C4	0.0526 (17)	0.0362 (16)	0.0461 (17)	0.0055 (13)	0.0243 (14)	0.0076 (13)
C5	0.0396 (15)	0.0417 (16)	0.0553 (18)	0.0098 (13)	0.0206 (14)	0.0113 (14)
C6	0.0401 (16)	0.0467 (17)	0.0552 (19)	0.0014 (14)	0.0213 (14)	0.0086 (15)
C7	0.0346 (15)	0.0460 (17)	0.0590 (19)	0.0066 (13)	0.0182 (14)	0.0092 (15)

### Geometric parameters (Å, °)

**Table:**

Co1—N1	2.020 (2)	N4—C2	1.381 (3)
Co1—N4	2.029 (2)	C1—H1	0.9300
Co1—Cl1	2.2478 (9)	C2—C3	1.347 (4)
Co1—Cl2	2.2676 (9)	C2—H2	0.9300
N1—C5	1.320 (3)	C3—H3	0.9300
N1—C6	1.385 (3)	C4—H4A	0.9700
N2—C5^i^	1.346 (3)	C4—H4B	0.9700
N2—C7	1.377 (3)	C5—N2^i^	1.346 (3)
N2—C4	1.464 (3)	C5—H5	0.9300
N3—C1	1.346 (3)	C6—C7^i^	1.349 (4)
N3—C3	1.369 (4)	C6—H6	0.9300
N3—C4	1.456 (3)	C7—C6^i^	1.349 (4)
N4—C1	1.317 (3)	C7—H7	0.9300
			
N1—Co1—N4	102.85 (9)	C3—C2—N4	109.3 (3)
N1—Co1—Cl1	114.03 (7)	C3—C2—H2	125.4
N4—Co1—Cl1	107.75 (7)	N4—C2—H2	125.4
N1—Co1—Cl2	109.58 (7)	C2—C3—N3	106.9 (2)
N4—Co1—Cl2	105.46 (7)	C2—C3—H3	126.6
Cl1—Co1—Cl2	115.95 (4)	N3—C3—H3	126.6
C5—N1—C6	105.2 (2)	N3—C4—N2	111.0 (2)
C5—N1—Co1	124.02 (18)	N3—C4—H4A	109.4
C6—N1—Co1	130.55 (18)	N2—C4—H4A	109.4
C5^i^—N2—C7	106.9 (2)	N3—C4—H4B	109.4
C5^i^—N2—C4	126.6 (2)	N2—C4—H4B	109.4
C7—N2—C4	126.4 (2)	H4A—C4—H4B	108.0
C1—N3—C3	106.9 (2)	N1—C5—N2^i^	111.7 (2)
C1—N3—C4	125.8 (2)	N1—C5—H5	124.1
C3—N3—C4	127.3 (2)	N2^i^—C5—H5	124.1
C1—N4—C2	105.6 (2)	C7^i^—C6—N1	109.8 (2)
C1—N4—Co1	125.08 (18)	C7^i^—C6—H6	125.1
C2—N4—Co1	129.31 (18)	N1—C6—H6	125.1
N4—C1—N3	111.4 (2)	C6^i^—C7—N2	106.4 (2)
N4—C1—H1	124.3	C6^i^—C7—H7	126.8
N3—C1—H1	124.3	N2—C7—H7	126.8
			
N4—Co1—N1—C5	1.3 (3)	C1—N4—C2—C3	0.6 (3)
Cl1—Co1—N1—C5	−115.1 (2)	Co1—N4—C2—C3	−178.2 (2)
Cl2—Co1—N1—C5	113.1 (2)	N4—C2—C3—N3	−1.2 (4)
N4—Co1—N1—C6	−172.0 (2)	C1—N3—C3—C2	1.3 (3)
Cl1—Co1—N1—C6	71.6 (3)	C4—N3—C3—C2	−179.8 (3)
Cl2—Co1—N1—C6	−60.2 (3)	C1—N3—C4—N2	−108.0 (3)
N1—Co1—N4—C1	88.2 (2)	C3—N3—C4—N2	73.3 (4)
Cl1—Co1—N4—C1	−151.0 (2)	C5^i^—N2—C4—N3	53.1 (4)
Cl2—Co1—N4—C1	−26.6 (2)	C7—N2—C4—N3	−130.9 (3)
N1—Co1—N4—C2	−93.2 (2)	C6—N1—C5—N2^i^	0.8 (3)
Cl1—Co1—N4—C2	27.6 (3)	Co1—N1—C5—N2^i^	−173.89 (18)
Cl2—Co1—N4—C2	152.0 (2)	C5—N1—C6—C7^i^	−0.5 (3)
C2—N4—C1—N3	0.3 (3)	Co1—N1—C6—C7^i^	173.8 (2)
Co1—N4—C1—N3	179.13 (17)	C5^i^—N2—C7—C6^i^	−0.5 (3)
C3—N3—C1—N4	−1.0 (3)	C4—N2—C7—C6^i^	−177.2 (3)
C4—N3—C1—N4	−179.9 (2)		

Symmetry codes: (i) −*x*+1, −*y*, −*z*+1.
